# The COVID-19 Pandemic: Public Health Responses in Sub-Saharan Africa

**DOI:** 10.3390/ijerph19084448

**Published:** 2022-04-07

**Authors:** Grant Murewanhema, Tafadzwa Dzinamarira

**Affiliations:** 1 Unit of Obstetrics and Gynaecology, Department of Primary Health Care Sciences, Faculty of Medicine and Health Sciences, University of Zimbabwe, Harare P.O. Box MP167, Zimbabwe; gmurewanhema@gmail.com; 2 ICAP at Columbia University, Harare P.O. Box MP167, Zimbabwe; 3 School of Health Systems & Public Health, University of Pretoria, Pretoria 0002, South Africa

**Keywords:** COVID-19, pandemic response, sub-Saharan Africa

## Abstract

The World Health Organisation declared the ongoing COVID-19 global health challenge a pandemic in March 2020. Since then, countries across the globe have implemented different public health control strategies—including global vaccination programs—in attempts to mitigate the further transmission of severe acute respiratory syndrome coronavirus 2. However, to date, the virus has continued to spread rapidly despite these interventions. Countries across sub-Saharan Africa have implemented variable control strategies to combat the pandemic; however, despite the continent being among the least affected in terms of direct case burden, morbidity, and mortality, it has experienced marked socioeconomic disruption. Therefore, economic resuscitation is an urgent priority. The continent is vastly underrepresented in the body of scientific evidence due to limited research resources, testing capacity and genomic surveillance leading to empirical responses or responses guided by evidence from elsewhere. To inform the ongoing pandemic, and to prepare for the future, this Special Issue calls for manuscripts on global COVID-19 responses, and encourages researchers and stakeholders from resource-limited settings, particularly from sub-Saharan Africa, to share their COVID-19 public health responses. Areas to be covered include, but are not limited to, surveillance, case management, infection prevention and control, risk communication and community engagement, logistics, laboratory, ports of entry, and co-ordination. Manuscripts including primary research, viewpoints/perspectives, and comprehensive literature reviews are all welcome.

The world has been battling the COVID-19 pandemic since the disease was declared a global pandemic in March 2020. Hence, we have reached the two-year mark of an ongoing pandemic that has significantly affected people’s livelihoods on a global scale, albeit to different extents. Most countries have gone through at least three to four periods of heightened transmission, known as waves, driven by different variants of concern, including the Alpha, Beta, Delta and Omicron variants. The World Health Organisation (WHO) epidemiological reports show that transmission of the aetiological severe acute respiratory syndrome coronavirus 2 has continued despite a myriad of public health interventions to limit its spread. As of 25 March 2022, the global cumulative number of cases reported is more than 476 million, with over 6,1 million fatalities, and nearly eight million incident cases in the preceding seven days [1]. At the time of writing, Africa has reported a cumulative 8,551,096 cases, 170,942 fatalities, and 20,249 incident cases in the preceding seven days [1]. Africa is one of the least affected continents in terms of direct morbidity, mortality, and absolute cumulative cases of COVID-19; however, the pandemic has been seriously disruptive to several socioeconomic aspects of the lives of African people. This Special Issue on the COVID-19 public health responses will allow researchers to share their experiences on what has and has not worked in terms of the COVID-19 responses of various countries. This information will be valuable for policymakers and other stakeholders to determine the most suitable steps forward in the ongoing pandemic, and inform future pandemic preparedness.

Countries across the world have implemented various control measures to combat the spread, morbidity, and mortality of the disease. Since this was a novel virus, most of the control measures were based on experiences from previous outbreaks of infectious diseases and pre-existing best practices. Fears of unprecedented mortality from COVID-19 resulted in some countries imposing extremely tough restriction measures on their populations, including a number of African countries that had very few or no reported cases of COVID-19 at the time. As an example, Zimbabwe imposed a national lockdown on the population on 30 March 2020, when only two cases of infection had been confirmed in the country [2]. The case burden in the country remained very low, with less than 1000 confirmed cases four months later. Contentiously, the reason for the low case burden has remained largely unknown—this cannot be attributed solely to the tough restriction measures. In an unprecedented achievement, by December 2020, some SARS-CoV-2 vaccines had emergency-use approvals by the WHO, and four months later, in April 2021, over one billion doses had been administered globally [1]. Not surprisingly, the majority of these were administered in developed countries, and most African countries still had to initiate their vaccination programs.

The initial responses across sub-Saharan Africa were very variable. Some countries, such as Rwanda, South Africa, Uganda, and Zimbabwe adopted very tough restrictions, banning local and international travel, closing industries, schools and universities, banning public transport, and restricting healthcare to ‘emergency mode’ [3]. Some of these countries adopted guidance from the WHO, stratifying their COVID-19 public health responses into several pillars including surveillance, case management, infection prevention and control, ports of entry, logistics, security, risk communication and community engagement, laboratory and security. The efficiency of such a guided response is still to be evaluated. On the other hand, some countries, such as Tanzania, decided upon a much more relaxed approach to pandemic, with the then political leaders of the country denouncing the existence of COVID-19 altogether [3]. As a result, there was no nationwide testing or surveillance of the disease in the country, and the damage this may have inflicted on the Tanzanian population remains unknown. Rwanda, South Africa and a number of other countries, on the other hand, took a very balanced approach to the pandemic, with phased lockdowns [4]. Notably, Rwanda was named among the top ten countries with the best response to the COVID-19 pandemic by the Lowy Institute in 2020 [5]. South Africa, in particular, has been commended for its efficient genomic surveillance leading to the early detection of variants of concern, the Beta and Omicron variants [6], a capacity which was generally limited or non-existent in other SSA countries without the support of international collaborating institutions.

The African continent was spared of severe morbidity and mortality in the initial wave of the pandemic; however, the Beta- and Delta-variant driven waves were destructive in some African countries, including Zimbabwe, Zambia, and South Africa, resulting in increased mortality and demand for healthcare services. These included admission space, high-dependency and intensive care, personal protective equipment, and healthcare personnel. The challenges occurred at a time when there was an increased demand for healthcare workers in the United Kingdom, the United States of America, and Europe, which resulted in the increased attrition of these essential personnel from Africa, leaving the continent disadvantaged and incapacitated to deal with its present and future healthcare needs. SSA urgently needs to step-up efforts to restore and maintain its healthcare workforce, including addressing the demands for better remuneration and conditions of service, and equipping facilities with modern equipment. African countries that have imposed repressive legislation on their healthcare workforce, such as Zimbabwe, have grossly failed to retain these essential personnel, instead pushing them away.

Countries in Europe, America, and Asia invested heavily in clinical and epidemiological research, and therefore have been able to formulate recent COVID-19 responses based on their own evidence. Unfortunately, in SSA, there has been a dearth of scientific studies, and the responses have continued to be empirical or guided by evidence from elsewhere [7]. This includes the safety and effectiveness of vaccines [8], including in vulnerable and specific populations such as children and pregnant women [9]. Genomic sequencing capacity, except in South Africa, is also generally limited on the continent; thus, delaying the detection of new variants of concern in circulation. However, substantial effort has been given since the arrival of COVID-19 to improve the continent’s sequencing capacity. One such example is the African Pathogen Genomics Initiative which includes centers in South Africa, Nigeria, Senegal, Ghana, Kenya, Democratic Republic of the Congo, and Uganda [10]. Additionally, a gross shortage of testing resources was common at the beginning of the pandemic, and the real-time polymerase chain reaction (RT-PCR) which was considered the gold standard, was expensive and inaccessible to many. Without adequate testing, surveillance was a challenge; particularly in the early stages of the pandemic, COVID-19 lacked a definitive diagnosis, with significant overlap with other acute respiratory conditions and great mimicry with a variety of other clinical conditions. With limited initial testing, the actual burden on the continent remains unknown [11]. Subsequently, countries have adopted other testing platforms such as the GeneXpert and rapid antigen tests, improving the availability and accessibility of testing and surveillance.

Two years into the pandemic, countries across the globe have forged ahead with SARS-CoV-2 vaccination programmes. Some countries, such as Seychelles, Mauritius, Morocco, and Rwanda have made significant progress, fully vaccinating almost 40% of their eligible populations, offering booster doses and extending vaccinations to school-going children [1]. On the other hand, other African countries such as Burundi, the Democratic Republic of Congo, and Chad have fallen significantly behind, and are yet to vaccinate at least 10% of their eligible populations [1]. Such inequitable distribution of vaccines provides a perfect, although unwelcome, opportunity for the continued emergence of variants of concern, and gives opportunity for breakthrough infections among the vaccinated [1]. Some vaccinated populations, such as those living with the human immunodeficiency virus (HIV) infection, are at a particularly high risk of breakthrough infections. The burden of HIV globally is highest in SSA [12]. It has been argued that as long as Africa relies on importing vaccines from the East and the West, the gap in vaccination will persist because of vaccine nationalism, and the hoarding of vaccines by richer countries. This has been seen with countries such as the United Kingdom pursuing booster dose administration ahead of African countries that are still yet to administer a single dose to substantial proportions of their populations. African countries must stop relying on Western governments and donor aid, place upon themselves the responsibility to provide quality healthcare for their populations, and invest adequately in local infrastructure to develop the ability to manufacture vaccines and other healthcare essentials.

The priority must now shift significantly towards living with the virus as vaccines become more widely available. SARS-CoV-2 vaccines have been shown to significantly reduce incident infection, symptomatic and severe disease, hospitalisations, intensive care unit admissions, and mortality from severe COVID-19. Socioeconomic restoration must be highly prioritised on the African continent to mitigate against further negative consequences on the population. This remains imperative despite the finding that Omicron has been found clinically to be less severe, and morbidity and mortality from this variant are much lower compared to previous variants. Priorities for the African continent must therefore be on providing vaccines to their populations, building local capacity for surveillance including genomic sequencing and vaccine manufacturing, and investing in local clinical and epidemiological research. Moving forward, responses to COVID-19 epidemic waves and any future public health emergencies must be based on locally generated evidence, as blanket measures such as the previous harsh lockdowns can be more damaging on the population than the emergency itself. Against this background, it is critical for African researchers and public health authorities to share experiences, previous and current, to guide both the present and the future. In this Special Issue, we focus on past and current COVID-19 experiences across the globe, and we encourage researchers from LMICs, and especially SSA, to share their experiences and learn from each other.
